# A Laser Line Auto-Scanning System for Underwater 3D Reconstruction

**DOI:** 10.3390/s16091534

**Published:** 2016-09-20

**Authors:** Shukai Chi, Zexiao Xie, Wenzhu Chen

**Affiliations:** Engineering College, Ocean University of China, 238 Songling Road, Qingdao 266100, China; chishukai@ouc.edu.cn (S.C.); 2002114@ouc.edu.cn (W.C.)

**Keywords:** underwater 3D reconstruction, laser line system, auto-scanning

## Abstract

In this study, a laser line auto-scanning system was designed to perform underwater close-range 3D reconstructions with high accuracy and resolution. The system changes the laser plane direction with a galvanometer to perform automatic scanning and obtain continuous laser strips for underwater 3D reconstruction. The system parameters were calibrated with the homography constraints between the target plane and image plane. A cost function was defined to optimize the galvanometer’s rotating axis equation. Compensation was carried out for the refraction of the incident and emitted light at the interface. The accuracy and the spatial measurement capability of the system were tested and analyzed with standard balls under laboratory underwater conditions, and the 3D surface reconstruction for a sealing cover of an underwater instrument was proved to be satisfactory.

## 1. Introduction

A large number of underwater applications require high resolution and accurate 3D reconstruction for underwater objects. The intervention tasks [1,2,3,4], pipes or other industrial facility inspection [5,6], archaeology [7,8,9,10], and biological applications [11,12] are just some examples. In these applications, sensors working at a short distance have to be used to obtain the 3D geometry information of an object accurately. These sensors can also increase the autonomy of underwater intervention systems. 

Compared with acoustic methods [13,14], optical methods with high resolution and accuracy are more suitable for short-distance operations [15]. The traditional passive vision systems are widely used because of their simplicity and low cost. Camera-based sensors are usually used as passive sensors since the other sensors are based on sounds or light projection. Passive methods sense the environment with a sequence of pictures taken from different viewpoints, so that 3D information will be recovered with the image features matching or stereo matching algorithms [16,17]. These systems are seriously affected by backscatter in limited visibility environments and need textured scenes to achieve satisfactory results [18,19]. As an active system, common structured light systems usually project a pattern onto the object with a projector to reconstruct [20]. The larger illumination volume and the projection light wavelength diversity lead to larger absorption and scattering coefficients. In underwater environments, laser-based structured light systems may be equipped with a different wavelength laser for the low absorption and scattering coefficients [21]. High brightness is generated with small power to improve the identification accuracy. Due to the small coverage area of a single laser stripe, a motion generator is often used to carry the laser to perform a scan. Such institutions usually exercise large size and power consumption [22,23].

The laser line scan (LLS) system was first used in [24] to reduce the backscattering. Nowadays, LLS systems have been used in various research fields such as underwater range finding [25], 3D reconstruction [26,27], and guidance of a robot in real time [28,29]. The laser line scan is performed with a handheld device [30], an electric motor [31], or a manipulator mounted on the robot [32,33]. There are also some commercial solutions available for 3D data gathering [34,35,36,37]. These systems can achieve high-resolution results with the precise control of the laser line movement, and they are more efficient than single laser line systems. However, the systems should be kept still while the laser line moves. The range of these systems does not exceed 3 m, even in clear water, normally because of the effects of absorption and scattering [38]. 

Our main motivation to develop this sensor was to provide visual guidance for an underwater intervention task with higher accuracy. This requires that the sensor can accurately measure the geometry information of the operation target, including shape, size, position, etc. The underwater vehicle-manipulator system (UVMS) is a typical platform for underwater intervention. Nowadays, few UVMSs can be considered to be able to perform autonomous manipulation. The underwater vehicle named SAUVIM (Semi Autonomous Underwater Vehicle for Intervention Mission, University of Hawaii) [3] hooked a cable under the guidance of a marker in the oceanic environment. A monocular vision system is used to detect the cable. Although the experiment focused on the integrity of the system, a monocular vision system is too simple to guide the robot to perform a more precise operation. The GIRONA500 [32] is an autonomous underwater vehicle developed in the university of Girona. It completed the reconstruction and grasping process on an amphora with a LLS mounted on the manipulator. The laser line swept across the object’s surface with the motion of the manipulator. The accuracy and resolution of the system were limited by the motion control accuracy of the manipulator. A LLS with a motion generator will be available as an improvement. Due to the sensor volume and consumption requirements of UVMSs and other underwater platforms, the motion generator should be small and efficient. In addition, the accuracy of the motion control is an important factor in the impact of 3D reconstruction. 

The system presented in this paper used a galvanometer as the motion mechanism. The laser plane direction was changed precisely by the rotation of the galvanometer. The control accuracy of the rotation angle of the galvanometer could reach 8 µrad, and this would provide a guarantee for the measurement accuracy and resolution. The outline dimension of the instrument was 580 mm (length) × 140 mm (width) × 205 mm (height), and the total weight in air was about 11.1 kg. The system operating voltage was 24 VDC, and the maximum power consumption was less than 7 W. The system with a smaller size and lower power consumption was suitable for installation onto an underwater mobile platform to perform underwater 3D reconstruction tasks.

This article is structured as follows: the description of the system construction is given in Section 2, the system calibration in Section 3, the compensation for the refraction caused by air-glass-water interface in Section 4, the experimental setup and results in Section 5, and the discussion and outlook in Section 6.

## 2. System Composition and Structure

**Figure 1:**
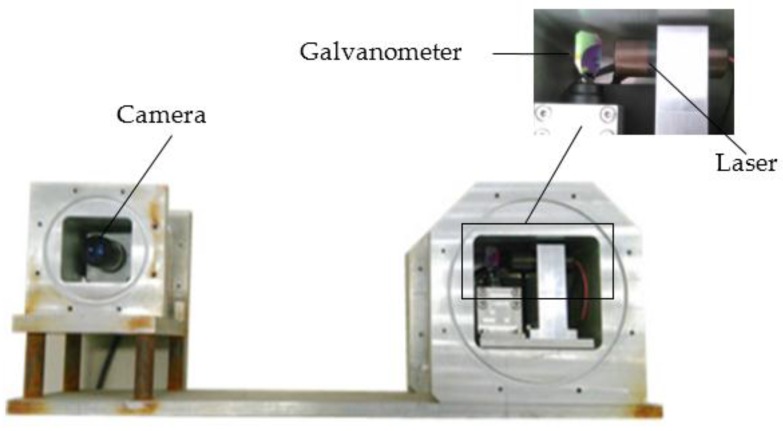
Laser line auto-scanning system.

As seen in Figure 1, a camera and a laser-galvanometer motion module unit were mounted in two independent watertight housings. The housings were made out of an aluminum alloy and were fixed to a base structure to keep its relative position stationary. The information such as the thickness of the glass and the distance between the camera and the sealed glass was accurately measured after the system was processed and installed. 

The WAT-902B (Watec Incorporated, Kawasaki, Japan) industrial-grade low-illumination analog camera and Computar 1214-MP (CBC Co., Ltd., Tokyo, Japan) megapixel lens produced by Watec were used. The galvanometer was a TS8720, manufactured by Sunny Technology (Beijing, China). The laser was LASIRIS series (ProPhotonix Limited, Salem, UT, USA). 

The camera CCD resolution was 752 (H) × 582 (V), the unit pixel size of image plane was 8.6 µm (H) × 8.3 µm (V), and the image resolution was 768 × 576. The galvanometer could be controlled to rotate in the range of ±20 by the voltage signal which ranges from −10 V to +10 V from a D/A conversion card in the computer.

After reflexed by the galvanometer, the laser plane was projected onto the object. The laser stripe moved with the galvanometer rotating in a certain step angle. The 3D data of the object surface could be obtained based on the computation of the optical information with the triangulation method.

## 3. System Calibration

**Figure 2:**
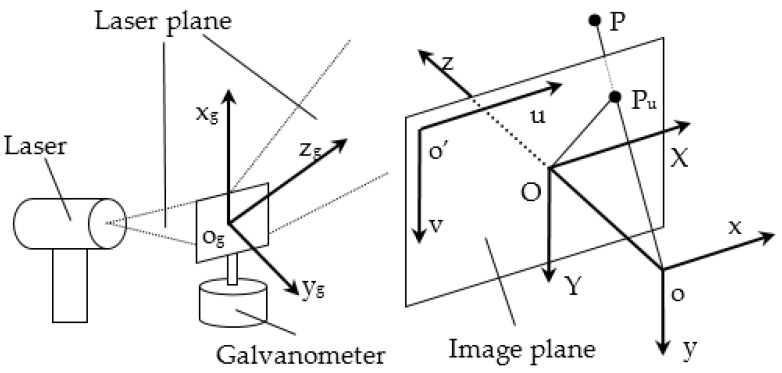
The schematic for system coordinate system setup.

The coordinate systems of the integrated system were set up as shown in Figure 2. The x_g_ axis is upward along the rotating axis of the galvanometer, o_g_ is the intersection point of the x_g_ axis and the o-yz plane of the camera coordinate system, and the y_g_ axis is outward perpendicular to the x_g_ axis from o_g_ in the exiting laser plane when the galvanometer control voltage is U_0_. The galvanometer rotates around the x_g_ axis, and the rotating angle could be controlled by the input voltage. In order to perform auto-scanning, the camera internal parameters and the transformation relation between the camera and the galvanometer were calibrated on land. The refraction caused by the air-glass-water interface was compensated when performing the underwater measurement.

**Figure 3:**
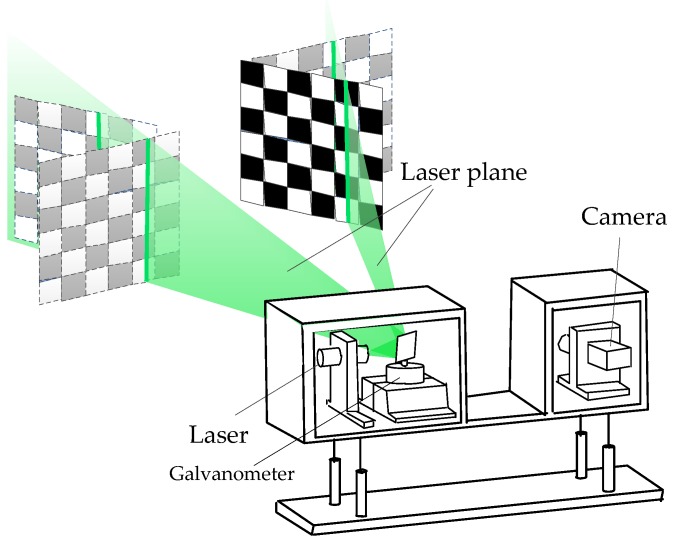
The schematic for the target location planning when performing the system calibration.

Calibration was performed with a planar checkerboard target. As seen in Figure 3, the calibration is completed by the following steps:

1. The target was placed in the measurement range to obtain a target image. Reasonable camera parameters such as gain and contrast are set to obtain the corresponding laser stripe image so that only the strongest laser spots were captured as saturated pixels. As the number of the points in the black squares has been already sufficient for fitting laser lines, the other points in the white squares could be ignored. This target image, together with the images obtained in the next two steps, were used to calibrate the camera internal parameters including the radial and decentering distortion parameters with the Zhang method [39]. The laser stripe image at the same position were used to calculate the coordinates in the camera coordinate system of the laser points with the rotation matrix and translation matrix, which were obtained when calibrating the camera internal parameters [40].
2. Keeping the laser plane emission angle constant, the position and the orientation of the target were changed multiple times in the view field of the system. Step 1 was then repeated. All laser spots obtained hitherto belonged to one laser plane, and these spots were used to fit the laser plane equations expressed in the camera coordinate system.
3. Laser plane emission angle was changed with the rotation of the galvanometer. Steps 1 and 2 were then repeated. Then, we obtained several different laser plane equations that could be used to calculate the galvanometer rotating axis equation.

The camera internal parameters were calibrated with the target images, and the transformation matrix between camera and galvanometer coordinates was calculated with the laser stripe images.

**Figure 4:**
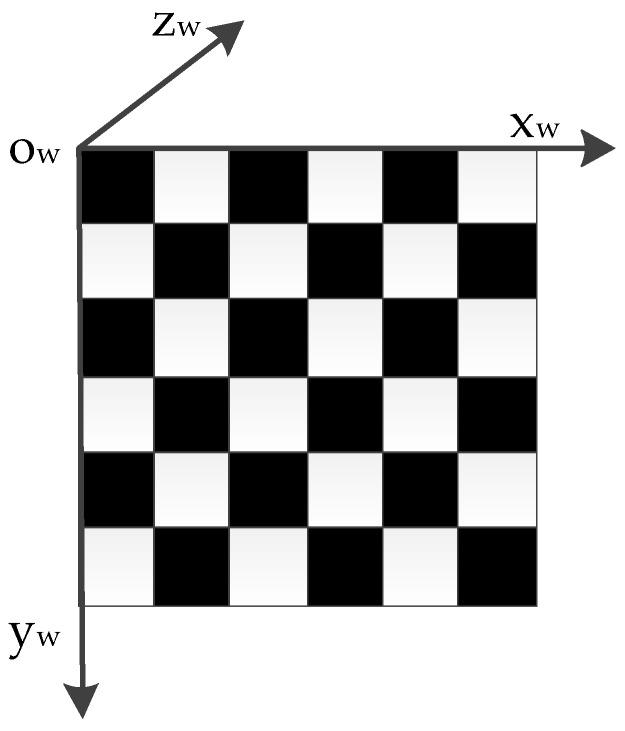
World coordinate system when performing camera calibration.

The world coordinate system was set up as shown in Figure 4 when the camera internal parameters were calculated, and this coordinate system is a right-handed system. According to the perspective projection principle, the relation between image plane coordinates and the world coordinates can be expressed as follows:

(1) $$\rho \left[\begin{array}{c} u\\ v\\ 1 \end{array}\right]=\left[\begin{array}{cccc} {\mathrm{fN}}_{x}r_{1}+r_{7}u_{0} & {\mathrm{fN}}_{x}r_{2}+r_{8}u_{0} & {\mathrm{fN}}_{x}r_{3}+r_{9}u_{0} & {\mathrm{fN}}_{x}t_{x}+t_{z}u_{0}\\ {\mathrm{fN}}_{y}r_{4}+r_{7}v_{0} & {\mathrm{fN}}_{y}r_{5}+r_{8}v_{0} & {\mathrm{fN}}_{y}r_{6}+r_{9}v_{0} & {\mathrm{fN}}_{y}t_{y}+t_{z}v_{0}\\ r_{7} & r_{8} & r_{9} & t_{z} \end{array}\right]\left[\begin{array}{c} x_{w}\\ y_{w}\\ z_{w}\\ 1 \end{array}\right],$$

where P (x_w_,y_w_,z_w_) in the world coordinate system images on the image plane is P_u_ (u,v). The camera internal parameters, including focal length f, the principal point coordinate (u_0_,v_0_), rotation matrix elements r_1_ to r_9_, and translation matrix elements t_x_, t_y_, t_z_. N_x_, and N_y_, were the number of pixels corresponding to the unit length on the image plane, respectively, and they are constants. These camera internal parameters and distortion parameters could be calculated using the Zhang calibration method. 

The laser plane was projected onto the target at different positions and orientations. The transformation relation between the camera coordinate system and the galvanometer coordinate system could be solved with the homography constraints. According to the definition of the world coordinate system shown in Figure 4, all laser points were on the target plane, so z_w_ = 0, and Equation (1) could be rewritten as Equation (2). The parameter matrix became a square matrix, so we could calculate the world coordinates with the known image plane coordinates.

(2) $$\rho \left[\begin{array}{c} u\\ v\\ 1 \end{array}\right]=\left[\begin{array}{ccc} {\mathrm{fN}}_{x}r_{1}{+r}_{7}u_{0} & {\mathrm{fN}}_{x}r_{2}{+r}_{8}u_{0} & {\mathrm{fN}}_{x}t_{x}+t_{z}u_{0}\\ {\mathrm{fN}}_{y}r_{4}{+r}_{7}v_{0} & {\mathrm{fN}}_{y}r_{5}{+r}_{8}v_{0} & {\mathrm{fN}}_{y}t_{y}+t_{z}v_{0}\\ r_{7} & r_{8} & t_{z} \end{array}\right]\left[\begin{array}{c} x_{w}\\ y_{w}\\ 1 \end{array}\right].$$

According to the rigid transformation relationship between the Euclidean coordinate systems, the transformation relationship between the camera coordinates and the world coordinates could be expressed as below:

(3) $$\left[\begin{array}{c} x\\ y\\ z \end{array}\right]=\left[\begin{array}{ccc} r_{1} & r_{2} & r_{3}\\ r_{4} & r_{5} & r_{6}\\ r_{7} & r_{8} & r_{9} \end{array}\right]\left[\begin{array}{c} x_{w}\\ y_{w}\\ z_{w} \end{array}\right]+\left[\begin{array}{c} t_{x}\\ t_{y}\\ t_{z} \end{array}\right].$$

The transformation matrices between the camera coordinate system and the world coordinate system when the position of the target was changed was obtained after calibrating the camera internal parameters. The coordinates of the laser points in the camera coordinate system could be obtained by solving Equations (2) and (3). All of the points in the same laser plane were used to solve the laser plane equation. This equation can be written as follows:

z = Ax + By + C.
(4)

Ideally, all light planes intersect in one line—the x_g_ axis. Due to the error caused by machining and assembly, the galvanometer rotating axis equation was optimized. The x_g_ axis equation in the camera coordinate system was defined as

(5) $$\{\begin{array}{c} \frac{{x-x}_{0}}{a}=\frac{{y-y}_{0}}{b}\\ \frac{{x-x}_{0}}{a}=\frac{{z-z}_{0}}{c} \end{array},$$

where (a,b,c) is the direction vector of x_g_ axis, (x_0_,y_0_,z_0_) is one point on the x_g_ axis. (x_0_,y_0_,z_0_) could be obtained by fitting all laser planes with the least square method. The normal vector of laser plane was perpendicular to the direction vector of the x_g_ axis according to the definition of the galvanometer coordinate system. Thus, the optimization function was defined with the dot product of the two vectors as the optimization goal.

(6) $$F=\overset{n}{\underset{i=1}{\sum }}{(j}_{i}{,k}_{i}{,l}_{i})\cdot (a,b,c),$$

where (j_i_,k_i_,l_i_) is the normal vector of the number i laser plane. F is optimized with the direction vector of the intersection of any two laser planes as the initial value to calculate the direction vector of the x_g_ axis. The direction vector of the z_g_ axis can be calculated with the galvanometer control voltage. The direction vector of y_g_ axis can be calculated with the direction vectors of x_g_ and y_g_ axis. The galvanometer coordinate system origin expression in camera coordinate system is $\left({0,\;y}_{0}-\frac{b}{a}x_{0}{,\;z}_{0}-\frac{b}{a}x_{0}\right)$.

The transformation relation between the camera coordinates and the galvanometer coordinates can be rewritten as Equation (7):

(7) $$\left[\begin{array}{c} x\\ y\\ z \end{array}\right]=\left[\begin{array}{ccc} r_{g1} & r_{g2} & r_{g3}\\ r_{g4} & r_{g5} & r_{g6}\\ r_{g7} & r_{g8} & r_{g9} \end{array}\right]\left[\begin{array}{c} x_{g}\\ y_{g}\\ z_{g} \end{array}\right]+\left[\begin{array}{c} t_{\mathrm{gx}}\\ t_{\mathrm{gy}}\\ t_{\mathrm{gz}} \end{array}\right]=\left[\begin{array}{ccc} x_{g} & y_{g} & z_{g}\\ 0 & 0 & 0\\ 0 & 0 & 0 \end{array}\begin{array}{ccc} 0 & 0 & 0\\ x_{g} & y_{g} & z_{g}\\ 0 & 0 & 0 \end{array}\begin{array}{ccc} 0 & 0 & 0\\ 0 & 0 & 0\\ x_{g} & y_{g} & z_{g} \end{array}\begin{array}{ccc} 1 & 0 & 0\\ 0 & 1 & 0\\ 0 & 0 & 1 \end{array}\right]M,$$

where M = [r_g1_…r_g9_,t_gx_,t_gy_,t_gz_]^T^. The galvanometer coordinate origin coordinates and the unit vectors of the three axes in Equation (7) were replaced to obtain the parameters matrix M.

According to the pinhole imaging model, we could obtain Equation (8).

(8) $$\{\begin{array}{c} \frac{z}{f}=\frac{x}{{(u-u}_{0}{)/N}_{x}}\\ \frac{z}{f}=\frac{y}{{(v-v}_{0}{)/N}_{y}} \end{array}.$$

The galvanometer coordinates of the object could be obtained with Equations (4), (7) and (8).

## 4. Compensation for the Refraction Caused by Air-Glass-Water Interface

Refraction caused by the air-glass-water interface results in high distortion on images when performing underwater measurement. Therefore, it was necessary to carry out refraction compensation for underwater measurements after calibration.

**Figure 5:**
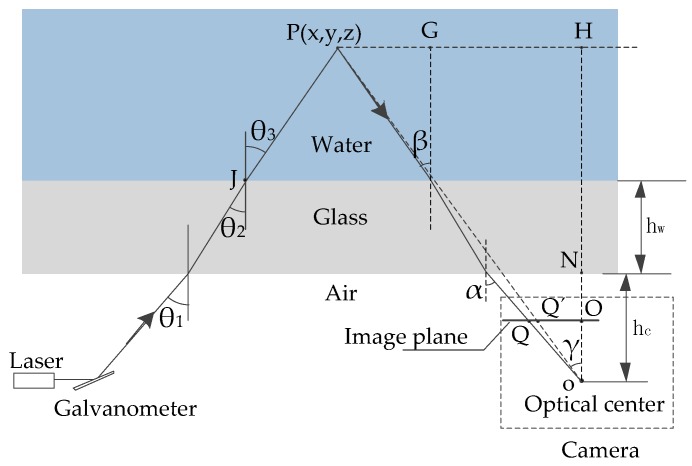
A planar view of the working system and the refraction caused by the air-glass-water interface of the incident and reflected light.

As shown in Figure 5, the incident laser projects onto the underwater object after reflecting at the medium conversion interface. According to Snell’s Law, Equation (9) could be obtained:

(9) $$\{\begin{array}{c} n_{a}{\sin\theta }_{1}=n_{g}{\sin\theta }_{2}\\ n_{g}{\sin\theta }_{2}=n_{w}{\sin\theta }_{3} \end{array}.$$

In this formula, θ_1_ could be calculated by the galvanometer deflection angle. n_a_, n_g_, and n_w_ are the refractive index in air, glass, and water, respectively. h_g_ and h_w_ are the distance from the rotating axis of the galvanometer to the sealed glass and the thickness of the glass, and they are known values. The coordinates of point J could be calculated via triangulation. The equation of the laser plane in air after reflexed by the galvanometer was then obtained. We then calculated the normal vector of the laser plane in water after refraction with the original normal vector and the coordinates of point J.

The underwater point P images on the camera image plane as point Q. If there were no air-glass-water interface refraction, the ideal imaging point would have been Q’. The relationship between the image point Q and Q’ image coordinates could be written as Equation (10):

(u’,v’) = k(u,v).
(10)

The k values of the different points on the image plane were different. According to the geometric relationship seen in Figure 5, we could obtain Equation (11):

(11) $$k=\frac{\mathrm{QO}}{Q’O}=\frac{\tan\gamma }{\tan\alpha }=\frac{\frac{\mathrm{NHtan}\beta +\mathrm{GH}}{\mathrm{oH}}}{\tan\alpha },\;\mathrm{and}$$

(12) $$\{\begin{array}{c} \mathrm{oO}=f\\ \mathrm{QO}=\sqrt{u^{2}{+v}^{2}}\\ \begin{array}{c} {\mathrm{oN}=h}_{c}\\ \mathrm{oH}=z \end{array} \end{array},$$

where the focal length of the camera f is obtained in the calibration process, and the image plane coordinates (u,v) is known. Substituting Equation (12) into Equation (11), we get Equation (13):

(13) $$k=\frac{\left(1-\frac{h_{c}}{z}\right)\tan\beta +\frac{h_{c}}{z}\tan\alpha }{\tan\alpha }.$$

Supposing that h_c_ << z, we can get a simplified expression for k:

(14) $$k\approx \frac{\tan\beta }{\tan\alpha }.$$

We could calculate the image plane coordinates of Q’ by triangulation with Equations (10) and (14). The image plane coordinates of Q’ were substituted into the equation of the laser plane after reflexed. Together with the measurement model on land, the 3D coordinates of the point P were obtained.

## 5. Experiments and Results

The experimental set-up in this article focused on the measurement feasibility and accuracy of the system. The system was deployed in a 1.5 m (length) × 0.9 m (width) × 0.9 m (height) stainless steel water tank. One side of the water tank was equipped with a 600 mm (width) × 600 mm (height) × 8 mm (thickness) glass window which made it convenient to observe.

**Figure 6:**
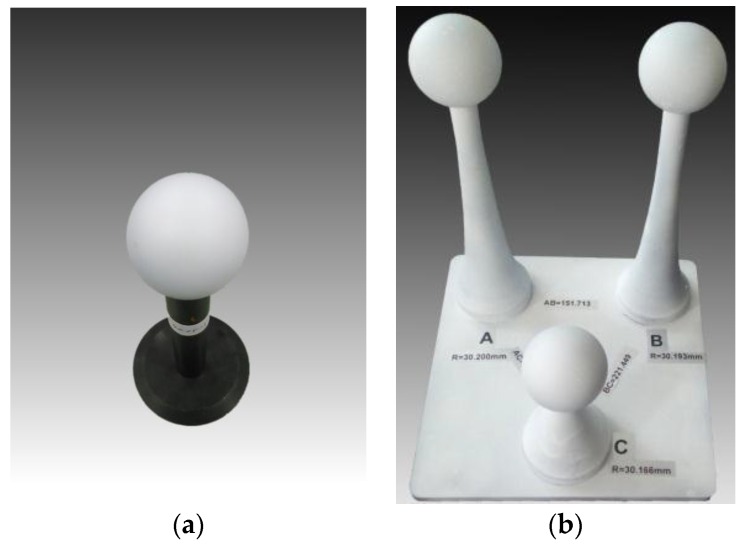
The balls that are already measured by a coordinate measurement machine (CMM) for the tank experiments. (**a**) The standard ball whose precise radius and spherical error are known for the accuracy test. (**b**) The three balls fixed on the one base board for the spatial error test. The radii of the three balls and the distances between any two balls are accurately known.

**Figure 7:**
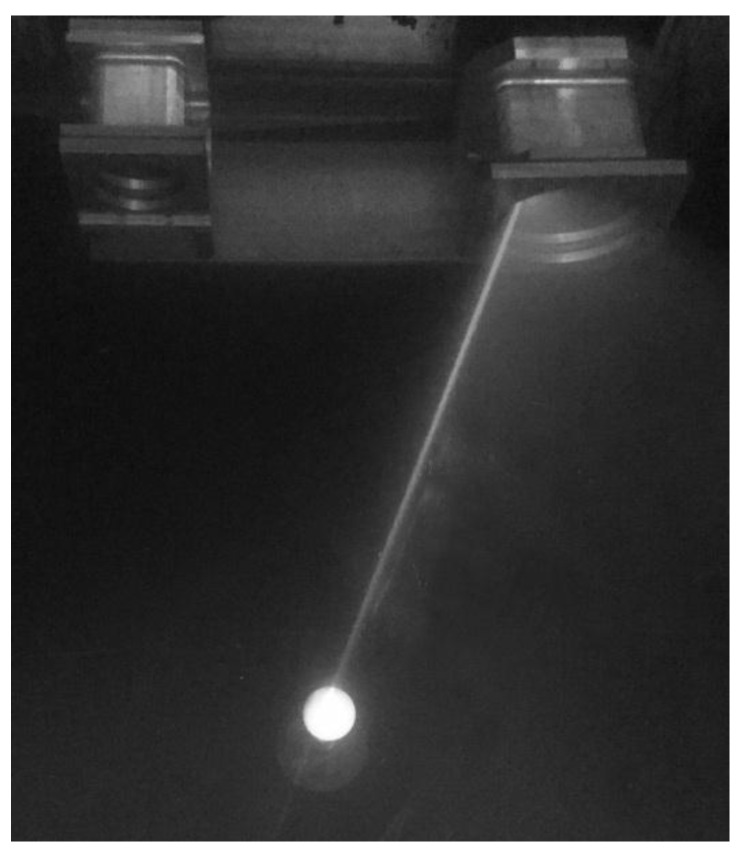
The standard ball measurement experimental setup in the water tank.

In this study, a ball as shown in Figure 6a was measured at different positions in the tank, as shown in Figure 7. The radius was 20.0175 mm, and the sphericity error was 0.0056 mm. The 3D information of the ball was obtained by fitting the point cloud with the commercial software NX Imageware Version 13.2 (Siemens PLM Software, Plano, America). The latest version of the software can be found at the company website [41]. The fitted radius value was compared with the actual value to evaluate the accuracy. The collected scatter points were used to analyze the measurement error distribution and stability. A three-ball system as seen in Figure 6b was used to assess the spatial error in the measurement field. A sealing cover of an underwater instrument was used to demonstrate the system’s ability to perform 3D reconstruction for general objects.

The z_g_ coordinates of the sphere center in the galvanometer coordinate system were along the system measurement depth direction. Thus, the z_g_ coordinate value of the ball represented the distance from the sphere center to the system. The ball was measured 10 times. As seen in Table 1, the z_g_ coordinate values of the sphere center in the 10 measurements ranged from 667.2722 to 1067.269. The number of points measured on the ball surface was reduced from 3635 to 1176 with increasing distance, and the resolution was high.

**Figure 8:**
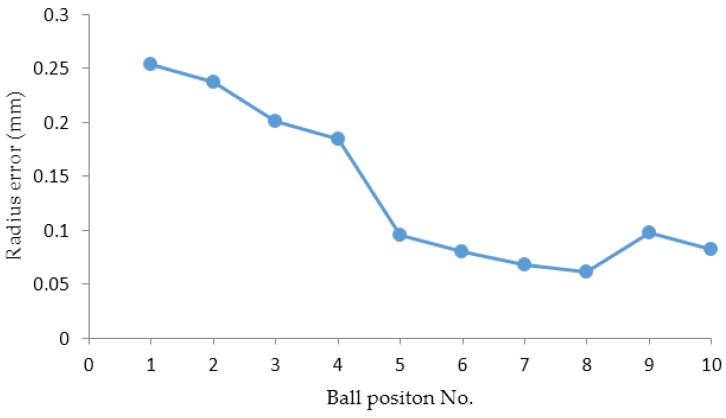
The errors between the fitted radii and the standard radius.

Figure 8 shows the errors between the standard radius and the fitted radii corresponding to the 10 positions. They were distributed from 0.0619 mm to 0.2537 mm. The errors became small and stable as the distance increased. This is because we assume h_c_ << z when compensating the interface refraction. Therefore, the error caused by the assumption became small when the z value became large. In experiments, the distance between the camera optical center and the glass surface was about 10 mm, and the z_g_ values of the ball ranged approximately from 667 mm to 1067 mm. 

**Figure 9:**
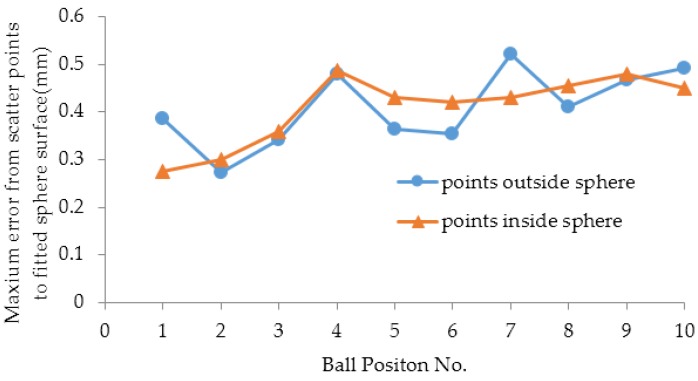
The maximum distance from the points outside and inside the sphere to the fitted surface.

The maximum distance from the points outside and inside the sphere to the fitted surface corresponding to 10 positions were respectively computed and are shown in Figure 9. They are randomly distributed without a regular trend.

**Figure 10:**
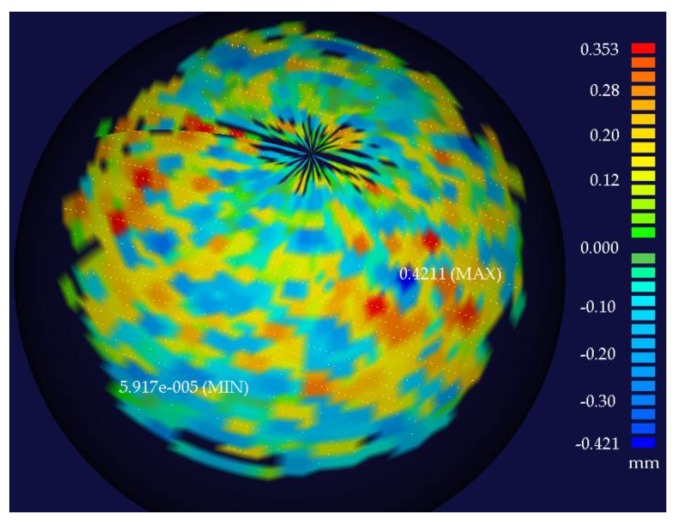
The error distribution between the fitted sphere and the scatter points measured at Position 6.

Figure 10 shows the error distribution between all of the scatter points and the fitted sphere surface measured at Position 6. The maximum distance from the points outside and inside the sphere to the fitted surface were 0.3532 mm and 0.4211 mm.

The three-ball system was measured 3 times with different orientations at the positions, which was about 1 m from the laser system. The three balls were marked as A, B, and C as seen in Figure 6b. AB, AC, and BC are the distances between the two corresponding balls’ centers. The width of the system’s view field at a position 1 m from the system was about 250 mm, and the three-ball system almost occupied the entire image in the experiments. The distances between any two balls were relatively long in the whole measurable volume and could be used to assess the spatial measurement errors. The results are in Table 2. The radius errors were distributed from −0.07 mm to 0.924 mm, and most of the errors were less than 0.5 mm. The maximum distance error value was 1.877 mm, and the most of the values were within 1 mm. The greater distance errors occurred when the distance directions approached the z_g_ axis direction. In addition to the affection of the assumption h_c_ << z on the foregoing, the errors of the processing and assembly parameters such as the distance between the galvanometer and glass also had a greater impact on the measurement error in the depth direction. 

**Figure 11:**
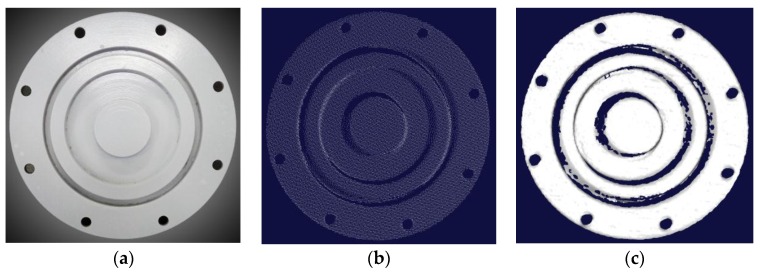
Measurement for a sealing cover of an underwater instrument. (**a**) Photograph; (**b**) Surface point cloud; (**c**) Triangular mesh representation.

A sealing cover of an underwater instrument was measured at a distance of about 1 m from the laser system. The outer ring radius of the cover was 78 mm. The galvanometer rotation angle increased from 25.49° to 38.37°, and the rotation step was 0.09°. 36127 points were obtained. As seen in Figure 11b,c, the point cloud was uniformly distributed with high resolution, and the triangular mesh model was smooth and complete.

In summary, under ideal laboratory conditions, the presented system was able to perform the 3D reconstruction for a smaller object at different positions with good accuracy. Moreover, the space measurement capability was thoroughly tested and analyzed with a three-ball system. By measuring a given cover, the system was proved to be effective in performing a 3D surface reconstruction with high resolution. In fact, accuracy and resolution were closely related to the working distance. T. Ekkel [42] used a laser line system to perform 3D measurement for welding seams at a closer distance (150 mm) with higher accuracy (35 µm). In [31], a device with a camera and motorized laser stripe was used to measure a board from 2 m away, and the accuracy was about 1 cm. 

## 6. Discussion and Outlook

The designed system extends measurement range with a galvanometer. The system can be equipped onto an underwater platform with a smaller size and lower power consumption, as well as a standalone sensor. 

The planning of the target positions in the calibration procedure is ingenious. In accordance with the planning, the laser points on the targets of different positions in the unified light plane will be obtained. The calibration is carried out with the coplanar constraint of these points. This greatly simplifies the calibration procedure and improves the efficiency. The rotation and translation matrices between the target and the camera coordinate systems in the calibration process of the camera's internal parameters can be directly used to perform the coordinates conversion of the laser points such that the computation cost is greatly reduced.

As seen in the results, the system has been proved to be effective in clear waters for close range static objects with high accuracy and resolution. The main source of the measuring error is the calibration error, and the following factors will cause the errors when performing actual measurement.

- System machining and assembly
  In this paper, we assume that the galvanometer rotating axis should completely coincide with the line intersected by the laser plane and the mirror of galvanometer. Assembly error will affect the accuracy of the measurement. The effect is reduced by the virtual axis with the optimization algorithm to improve the accuracy in the actual operation. In addition, the other machining and assembly parameters can also lead to the calculation error of the triangulation.
- Parameters of the camera and laser
  An external environment with a different light will cause the contrast and definition difference of the image. The camera gain, offset, and contrast parameters as well as the laser intensity should be adjusted accordingly to improve optical quality so as to improve the accuracy. The results are stable in dark environments.
- The angle between light plane and spherical surface
  When the angle is small, the width of the light strip formed by the laser line and spherical intersecting becomes large and the length becomes short. All of the above have a great influence on the extraction of the light stripe center.

The results demonstrate the accuracy under ideal conditions. In application, measurement results will be affected by environmental factors such as turbidity, illumination, salinity, current, etc. All of these factors should be considered in future experimental settings. More realistic experiments in practical situations (i.e., sea water conditions) will comprise our future work. Comparisons between measurements with different levels of turbidity and illumination will be performed to analyze the influence on the accuracy and robustness. In general, the effects caused by turbidity is acceptable [27]. Light scattering is increased and the image definition is degraded. A larger power laser may be a solution. 

At present, the system in this paper can perform underwater scanning and obtain a 3D point cloud. All radii and distance data in the experiments were obtained with commercial software fitting. An algorithm that can automatically separate rule shape objects from the view field point cloud and perform the surface shape and fitting is under development and experimental testing. The algorithm focus on general objects with irregular shapes will also be considered. 

## Figures and Tables

**Table 1:** The 10 fitted sphere centers galvanometer coordinates, radii (mm), and detected points numbers.

Ball Position No.	Fitted Sphere Centers			Fitted Radii	Points Number
	x_g_	y_g_	z_g_		
1	18.6293	−71.9997	667.2722	20.2712	3635
2	18.6899	−79.499	721.507	20.2547	2978
3	19.5748	−39.5651	772.5898	20.2187	2870
4	19.7713	−39.3427	817.5533	20.2018	2380
5	19.8753	−29.6658	858.9471	20.1116	1958
6	19.9782	−23.9347	912.6583	20.0975	2017
7	20.3525	−16.948	958.4314	20.0857	1701
8	21.1155	−23.5845	998.197	20.0794	1527
9	21.6605	−32.4656	1021.3342	20.1154	1433
10	23.3907	−37.6898	1067.269	20.1003	1176

**Table 2:** Summary of the fitted radii and distances values of the three-ball system. The errors between the fitted values and the standard values are also listed.

Position No.	Value Type	Radii (mm)			Distances (mm)		
		A	B	C	AB	AC	BC
-	Standard Values	30.200	30.193	30.166	151.713	220.056	221.449
1	Measured Values	30.612	30.939	31.089	151.682	219.197	222.09
	Errors	0.412	0.746	0.923	−0.031	−0.859	0.641
2	Measured Values	29.968	30.461	30.631	150.699	218.179	220.716
	Errors	−0.232	0.268	0.465	−1.014	−1.877	−0.733
3	Measured Values	30.133	30.245	30.519	150.694	219.372	222.438
	Errors	−0.067	0.052	0.353	−1.019	−0.684	0.989
